# Impact of Fibronectin Knockout on Proliferation and Differentiation of Human Infrapatellar Fat Pad-Derived Stem Cells

**DOI:** 10.3389/fbioe.2019.00321

**Published:** 2019-11-15

**Authors:** Yiming Wang, Yawen Fu, Zuoqin Yan, Xiao-Bing Zhang, Ming Pei

**Affiliations:** ^1^Stem Cell and Tissue Engineering Laboratory, Department of Orthopaedics, West Virginia University, Morgantown, WV, United States; ^2^Department of Orthopaedics, Zhongshan Hospital, Fudan University, Shanghai, China; ^3^State Key Laboratory of Experimental Hematology, Institute of Hematology and Blood Disease Hospital, Tianjin, China; ^4^Department of Medicine, Loma Linda University, Loma Linda, CA, United States; ^5^WVU Cancer Institute, Robert C. Byrd Health Sciences Center, West Virginia University, Morgantown, WV, United States

**Keywords:** fibronectin, infrapatellar fat pad-derived stem cell, proliferation, chondrogenic differentiation, adipogenic differentiation

## Abstract

Fibronectin plays an essential role in tissue development and regeneration. However, the effects of fibronectin knockout (FN1-KO) on stem cells' proliferation and differentiation remain unknown. In this study, CRISPR/Cas9 generated FN1-KO in human infrapatellar fat pad-derived stem cells (IPFSCs) was evaluated for proliferation ability including cell cycle and surface markers as well as stemness gene expression and for differentiation capacity including chondrogenic and adipogenic differentiation. High passage IPFSCs were also evaluated for proliferation and differentiation capacity after expansion on decellularized ECM (dECM) deposited by FN1-KO cells. Successful FN1-KO in IPFSCs was confirmed by Sanger sequencing and Inference of CRISPR Edits analysis (ICE) as well as immunostaining for fibronectin expression. Compared to the GFP control, FN1-KO cells showed an increase in cell growth, percentage of cells in the S and G_2_ phases, and CD105 and CD146 expression but a decrease in expression of stemness markers CD73, CD90, SSEA4, and mesenchymal condensation marker *CDH2* gene. FN1-KO decreased both chondrogenic and adipogenic differentiation capacity. Interestingly, IPFSCs grown on dECMs deposited by FN1-KO cells exhibited a decrease in cell proliferation along with a decline in *CDH2* expression. After induction, IPFSCs plated on dECMs deposited by FN1-KO cells also displayed decreased expression of both chondrogenic and adipogenic capacity. We concluded that FN1-KO increased human IPFSCs' proliferation capacity; however, this capacity was reversed after expansion on dECM deposited by FN1-KO cells. Significance of fibronectin in chondrogenic and adipogenic differentiation was demonstrated in both FN1-KO IPFSCs and FN(–) matrix microenvironment.

## Introduction

As a connective tissue, articular cartilage is susceptible to damage caused by trauma or osteoarthritis (OA). However, its healing response to injury is limited due to its avascular nature (Benedek, 2006). Despite many pre-clinical studies having been performed, the regeneration of functional articular cartilage for clinical use remains a challenge (Karnes et al., 2014). An increasing body of evidence indicates that mesenchymal stem cells (MSCs) have great potential for cartilage engineering and regeneration (Jones and Pei, 2012; Pizzute et al., 2015). After first being isolated from bone marrow (Friedenstein et al., 1976), MSCs have been found in a variety of tissues including infrapatellar fat pad (IPFP) (Sun et al., 2018a). IPFP-derived MSCs (IPFSCs) are easily accessible and have better chondrogenic potential than bone marrow-derived MSCs (BMSCs) (Hindle et al., 2017). IPFSCs from OA patients were shown to possess comparable chondrogenic potential as those from non-OA donors (Liu et al., 2014), supporting the feasibility of using patients' autologous cells for regeneration. However, MSCs including IPFSCs were reported to inevitably suffer from cell senescence due to *in vitro* expansion or donor age (Li and Pei, 2012; Lynch and Pei, 2014).

Recent studies indicate that microenvironment, provided by extracellular matrix (ECM), plays an important role in the regulation of stem cell stemness (Pei, 2017; Sun et al., 2018b). For instance, decellularized ECM (dECM) has been demonstrated to rejuvenate human IPFSCs (He and Pei, 2013), synovium-derived MSCs (SDSCs) (Li et al., 2014), and human BMSCs (Pei et al., 2011a). Fibronectin (FN), one of the major fibrillary components in ECM, is implicated in the proliferation and differentiation processes of MSCs (Chang et al., 2008; Kalkreuth et al., 2014). However, while most evidence relies on the effect of fibronectin ligands on cell behavior (Linask and Lash, 1988; Budd et al., 1990; Sapudom et al., 2015), with a few reports investigating the effect *via* fibronectin knockout (FN1-KO) (Liu et al., 2010; Lukjanenko et al., 2016), there is no evidence of the impact of FN1-KO on adult stem cells' chondrogenic capacity. Therefore, in this study, the FN1-KO approach was used to investigate the role of fibronectin in guiding IPFSCs' chondrogenic and adipogenic differentiation given the close relationship between these two lineages (Zhou et al., 2019) and in this specific type of stem cells (Sun et al., 2018a). Furthermore, the role of fibronectin on IPFSCs' proliferation and bi-lineage differentiation was evaluated *via* dECM deposited by FN1-KO IPFSCs, in other words, a three-dimensional FN(–) matrix microenvironment.

## Materials and Methods

### IPFSC Harvest and Culture

Approval for this study was obtained from the Institutional Review Board. Human adult IPFPs were harvested from six young patients with acute meniscus or anterior crucial ligament tear (four male and two female, average 22 years old). These IPFPs were minced and sequentially digested with 0.1% trypsin (Roche, Indianapolis, IN) for 30 min and 0.1% collagenase P (Roche) for 2 h to separate cells. After filtration and centrifugation, obtained IPFSCs were pooled and cultured in growth medium [Minimum Essential Medium–Alpha Modification (αMEM) containing 10% fetal bovine serum (FBS), 100 U/ml penicillin, 100 μg/ml streptomycin, and 0.25 μg/ml fungizone (Invitrogen, Carlsbad, CA)] at 37°C in a humidified 21% O_2_ and 5% CO_2_ incubator. The medium was changed every 3 days.

### Single-Guide RNA (sgRNA) Design, Plasmid Construction, and Virus Production

The CHOPCHOP website (https://chopchop.rc.fas.harvard.edu/) was consulted to design high-performance sgRNAs targeting FN1 (Zhang et al., 2016) sgFN1a (GCTGTAACCCAGACTTACGG) and sgFN1b (GCAAGCGTGAGTACTGACCG) were used in this study. Lentiviral vectors that express Cas9 (driven by the SFFV promoter) and sgRNA (driven by the U6 promoter) were constructed with a NEBuilder HiFi DNA Assembly Kit (New England Biolabs, Ipswich, MA). The vectors were verified by Sanger sequencing of the inserts. A standard calcium phosphate precipitation protocol was utilized for lentivirus production. The lentiviral vectors were condensed 100-fold by centrifugation at 6,000 × *g* for 24 h at 4°C to reach biological titers of ~1 × 10 (Hindle et al., 2017)/ml.

### Lentiviral CRISPR/Cas9 Mediated FN1-KO

Lentiviral CRISPR/Cas9 was used to generate FN1-KO in human IPFSCs according to a previous report (Zhang et al., 2017). Passage 1 human IPFSCs were transduced at a multiplicity of infection (MOI) of two with scramble sgRNA sequence-containing vector (green fluorescence protein control lentivirus particles, copGFP) or CRISPR/Cas9 vectors (sgFN1a and sgFN1b) in the presence of 4 μg/ml of protamine sulfate (MilliporeSigma, Burlington, MA). After 24 h, the medium was changed to αMEM with 10% FBS and 2 μg/ml of puromycin (MilliporeSigma) for selection. Five days after transduction and puromycin selection, DNA fragments surrounding the Cas9-sgRNA target sites were polymerase chain reaction (PCR) amplified. Sanger sequencing and Inference of CRISPR Edits (ICE) were used to evaluate the frameshift-induced knockout efficiency (Li et al., 2018). Meanwhile, immunofluorescence staining for fibronectin was also used to confirm transduction efficiency in the dECMs deposited by normal cells (normal ECM), Cas9-sgFN1a transduced cells (sgFN1a ECM), and Cas9-sgFN1b transduced cells (sgFN1b ECM).

### dECM Preparation and Immunofluorescence Staining

The protocol to prepare dECM was detailed in a previous report (Li and Pei, 2018). Briefly, tissue culture plastic (TCP) was pre-coated with 0.2% gelatin (MilliporeSigma) at 37°C for 1 h, followed by treatment with 1% glutaraldehyde (MilliporeSigma) and 1 M ethanolamine (MilliporeSigma) at room temperature (RT) for 0.5 h, respectively. Passage 5 IPFSCs from the copGFP, sgFN1a, and sgFN1b groups were seeded on pre-coated TCP (6,000 cells/cm^2^) until they reached 100% confluence, followed by addition of L-ascorbic acid phosphate (Wako Chemicals, Richmond, VA) in the medium at a working concentration of 250 μM for an additional 7 days (Pizzute et al., 2016). Then, cells were incubated in 0.5% Triton X-100 (MilliporeSigma) containing 20 mM ammonium hydroxide (Sargent-Welch, Skokie, IL) at 37°C for 5 min. After the cells were removed, dECMs were rinsed with phosphate buffered solution (PBS) and stored in PBS containing 100 U/ml penicillin, 100 μg/ml streptomycin, and 0.25 μg/ml fungizone at 4°C until use.

dECMs were fixed in 4% paraformaldehyde, blocked with 1% bovine serum albumin (BSA), and incubated with primary antibody against human fibronectin (cat no. HFN 7.1; Developmental Studies Hybridoma Bank, Iowa City, IA). After rinsing with PBS, dECMs were incubated with secondary antibody [Donkey anti-Mouse IgG (H+L) Alexa Fluor 488, Invitrogen]. Fluorescence intensity was observed under a Zeiss Axiovert 40 CFL Inverted Microscope (Zeiss, Oberkochen, Germany).

### Culture of IPFSCs on TCP and dECMs

Two experiments were designed as follows: (1) TCP culture regimen (Experiment 1), passage 5 IPFSCs from the copGFP, sgFN1a, and sgFN1b groups were expanded on TCP; and (2) dECM culture regimen (Experiment 2), high passage (passage 15) IPFSCs were expanded for 7 days on TCP and dECMs deposited by passage 5 IPFSCs from the copGFP, sgFN1a, and sgFN1b groups in terms of copGFP ECM, sgFN1a ECM, and sgFN1b ECM. Expanded cells were detached followed by incubation in a pellet culture system for chondrogenic induction or culture in T25 flasks for adipogenic induction.

### Evaluation of Expanded Cells' Growth Rate, Surface Phenotypes, and Expression of Stemness Genes

Cell number was counted (*n* = 8 T175 flasks each group) and cell cycle was measured (the percentage of cells in the S and G_2_ phases) to assess expanded cell growth. After a 7-day culture of seeded cells at 3,000 cells/cm^2^, the harvested cells were counted using Countess® (Invitrogen). For cell cycle analysis, cells were fixed with 70% ethanol and stained with propidium iodide (MilliporeSigma). DNA contents were measured using FACS Calibur (BD Biosciences, San Jose, CA), and analyzed using FCS Express software package (*De Novo* Software, Los Angeles, CA).

Flow cytometry was used to evaluate surface phenotypes of expanded cells. The following primary antibodies were used: CD73-APC (cat no. 17-0739-42; eBioScience, Fisher Scientific, Waltham, MA), CD90-APC-Vio770 (cat no. 130-114-863; Miltenyi Biotec, San Diego, CA), CD105-PerCP-Vio700 (cat no. 130-112-170; Miltenyi Biotec), CD146-PE (cat no. 12-1469-42; eBioScience), and the stage-specific embryonic antigen 4-PE (SSEA4-PE; cat no. 330406; BioLegend, Dedham, MA). Samples of each 2 × 10^5^ expanded cells were incubated in cold PBS containing 0.1% ChromPure Human IgG whole molecule (Jackson ImmunoResearch Laboratories, West Grove, PA) for 30 min, followed by binding with the primary antibodies at 4°C for 30 min. Fluorescence was examined by a FACS Calibur (BD Biosciences) using FCS Express software package (*De Novo* Software).

Total RNA was extracted from expanded cells (*n* = 4) using an RNase-free TRIzol® (Invitrogen). About 2 μg of mRNA was utilized for reverse transcription with a High-Capacity cDNA Reverse Transcription Kit (Applied Biosystems Inc., Foster, CA). Stemness genes [*NANOG* (assay ID: Hs02387400_g1), *SOX2* (SRY-box 2; assay ID: Hs01053049_s1), *KLF4* (Kruppel-like factor 4; assay ID: Hs00358836_m1), *BMI1* (B lymphoma Mo-MLV insertion region 1 homolog; assay ID: Hs00180411_m1), *MYC* (assay ID: Hs00153408_m1), *NOV* (nephroblastoma overexpressed; assay ID: Hs00159631_m1), *POU5F1* (POU class 5 homeobox 1; assay ID: Hs04260367_gH), and *NES* (nestin; assay ID: Hs04187831_g1)], senescent genes [*CDKN1A* (cyclin-dependent kinase inhibitor 1A; assay ID: Hs00355782_m1), *CDKN2A* (cyclin-dependent kinase inhibitor 2A; assay ID: Hs00923894_m1), and *TP53* (tumor protein p53; assay ID: Hs01034249_m1)], and the mesenchymal condensation gene [*CDH2* (cadherin 2; assay ID: Hs00983056_m1)] were customized by Applied Biosystems as part of the Custom TaqMan® Gene Expression Assays. *GAPDH* (glyceraldehyde-3-phosphate dehydrogenase; assay ID: Hs02758991_g1) was used as the endogenous control gene. Real-time quantitative PCR (qPCR) was performed using Applied Biosystems™ 7500 Fast Real-Time PCR System (Applied Biosystems). Relative transcript levels were calculated as χ = 2^−ΔΔCt^, in which ΔΔCt = ΔE – ΔC, ΔE = Ct_exp_ – Ct_GAPDH_, and ΔC = Ct_ct1_-Ct_GAPDH_.

### Chondrogenic Induction and Analysis

For chondrogenic induction, aliquots of 0.3 × 10^6^ expanded cells were centrifuged at 500 *g* for 7 min in a 15-ml polypropylene tube to make a pellet. After overnight incubation (day 0), pellets were grown in a serum-free chondrogenic induction medium [high-glucose Dulbecco's modified Eagle's medium (DMEM) with 40 μg/ml proline (MilliporeSigma), 100 nM dexamethasone (MilliporeSigma), 100 U/ml penicillin, 100 μg/ml streptomycin, 0.1 mM ascorbic acid-2-phosphate, and 1 × ITS™ Premix (BD Biosciences)] with the supplementation of 10 ng/ml transforming growth factor beta3 (TGFβ3; PeproTech, Rocky Hill, NJ) for up to 18 days. Chondrogenic differentiation was assessed using histology, immunohistochemistry, and qPCR.

Representative pellets (*n* = 3) were fixed in 4% paraformaldehyde at 4°C overnight, followed by dehydrating in a gradient ethanol series, clearing with xylene, and embedding in paraffin blocks. Five-micrometer-thick sections were stained with Alcian blue (MilliporeSigma) staining for sulfated glycosaminoglycan (GAG). For immunohistochemical staining (IHC), consecutive sections were incubated with primary antibody against type II collagen (cat no. II-II6B3; Developmental Studies Hybridoma Bank) followed by the secondary antibody of biotinylated horse anti-mouse IgG (Vector, Burlingame, CA). Immunoactivity was identified using Vectastain ABC reagent (Vector).

Total RNA was extracted from chondrogenically induced pellets (*n* = 4) using an RNase-free TRIzol® (Invitrogen). After reverse transcription, chondrogenic marker-related genes [*SOX9* (SRY-box 9; assay ID: Hs00165814_m1), *ACAN* (aggrecan; assay ID: Hs00153936_m1), *COL2A1* (type II collagen; assay ID: Hs00156568_m1), and *PRG4* (proteoglycan 4; assay ID: Hs00981633_m1)] and hypertrophic marker genes [*COL10A1* (type X collagen; assay ID: Hs00166657_m1) and *MMP13* (matrix metallopeptidase 13; assay ID: Hs00233992_m1)] were customized by Applied Biosystems as part of the Custom TaqMan® Gene Expression Assays. *GAPDH* was used as the endogenous control gene. Each experiment was repeated three times.

### Adipogenic Induction and Analysis

When cells reached 90% confluence in T25 flasks, they were cultured for 21 days in adipogenic medium (growth medium supplemented with 1 μM dexamethasone, 0.5 mM isobutyl-1-methyxanthine, 200 μM indomethacin, and 10 μM insulin). Cells in T25 flasks (*n* = 3) were fixed in 4% paraformaldehyde and stained with a 0.6% (w/v) Oil Red O (ORO) solution (60% isopropanol, 40% water) for 10 min. Intracellular lipid-filled droplet-bound staining was recorded under a Nikon TE300 phase-contrast microscope (Nikon, Tokyo, Japan).

Total RNA was extracted from adipogenically induced cells (*n* = 4) using an RNase-free TRIzol® (Invitrogen). After reverse transcription, adipogenic marker genes [*LPL* (lipoprotein lipase; assay ID: Hs00173425_m1), *PPARG* (peroxisome proliferator-activated receptor gamma; assay ID: Hs01115513_m1), *FABP4* (fatty acid-binding protein 4; assay ID: Hs01086177_m1), and *CEBPA* (CCAAT enhancer binding protein alpha; assay ID: Hs00269972_s1)] were customized by Applied Biosystems as part of the Custom TaqMan® Gene Expression Assays. *GAPDH* was used as the endogenous control gene. Each experiment was repeated three times.

### Statistical Analysis

Mann–Whitney *U* test was used for pairwise comparison. All statistical analyses were conducted with SPSS 20.0 statistical software (SPSS Inc., Chicago, IL). *P* < 0.05 was considered statistically significant.

## Results

### FN1-KO Cell Model and Influence on IPFSCs in Proliferation and Stemness

**Figure 1:**
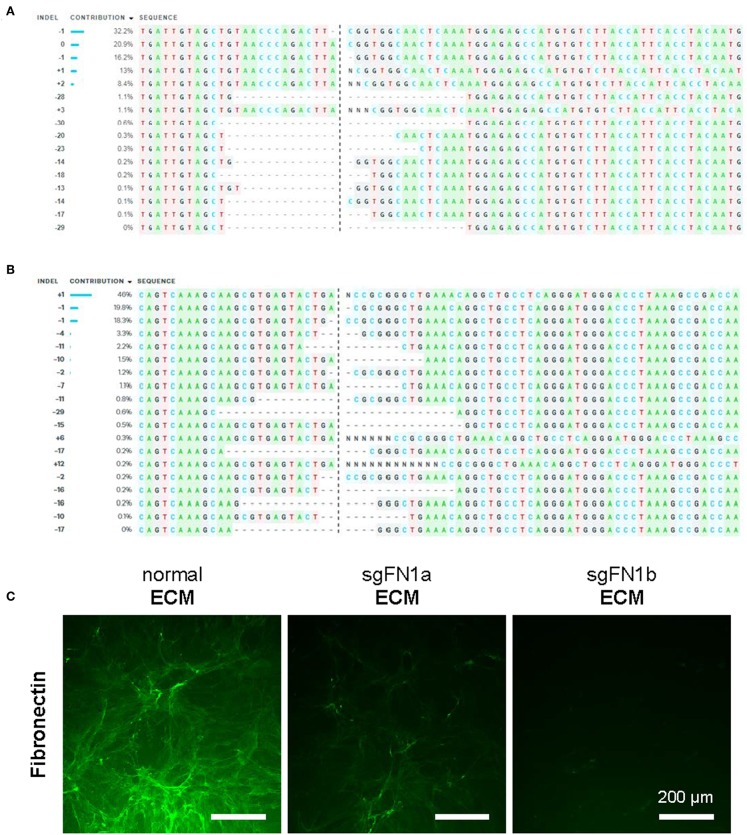
Knockout of FN1 in IPFSCs. Human IPFSCs were transduced with scramble sgRNA sequence-containing vector (green fluorescence protein control lentivirus particles, copGFP) or CRISPR/Cas9 vectors (sgFN1a and sgFN1b). Five days after transduction, amplicons targeting the Cas9-sgFN1 cleavage sites were subject to Sanger sequencing and ICE analysis. Representative diagrams of indel mutations were induced by sgFN1a **(A)** and sgFN1b **(B)**. FN1-KO was also confirmed by immunofluorescence staining for fibronectin in the dECMs deposited by normal cells (normal ECM) and Cas9-sgFN1a/b transduced cells (sgFN1a ECM and sgFN1b ECM, respectively) **(C)**.

In this study, CRISPR/Cas9 was used to generate knockout FN1 in human IPFSCs. To confirm the success of FN1-KO, Sanger sequencing and ICE analysis were conducted and the results revealed 74% indels for sgFN1a (Figure 1A) and 96% indels for sgFN1b (Figure 1B). This result was in line with our immunofluorescence data (Figure 1C). We found that, compared to abundant expression of fibronectin in “normal ECM” deposited by non-transduced IPFSCs, “sgFN1a ECM” and “sgFN1b ECM” exhibited considerably less expression of fibronectin, particularly for the sgFN1b ECM group.

**Figure 2:**
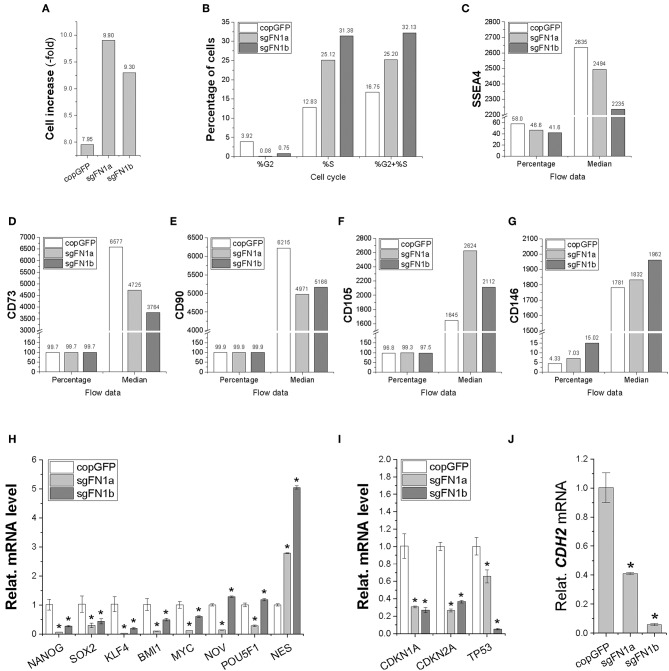
Cell proliferation capacity in human IPFSCs after FN1-KO. FN1-KO cells were compared with copGFP in cell increase **(A)**, percentage of cells in the S and G_2_ phases **(B)**, and surface markers [SSEA4 **(C)**, CD73 **(D)**, CD90 **(E)**, CD105 **(F)**, and CD146 **(G)**] by flow cytometry; stemness genes (*NANOG, SOX2, KLF4, BMI1, MYC, NOV, POU5F1*, and *NES*) **(H)**, senescent genes (*CDKN1A, CDKN2A*, and *TP53*) **(I)**, and the mesenchymal condensation gene (*CDH2*) **(J)** by qPCR. *GAPDH* was used as an endogenous control. Data are shown as bar charts. * indicates a significant difference compared to the corresponding copGFP group (*P* < 0.05).

In order to determine whether fibronectin influences stem cell proliferation, cell increase was measured (Figure 2A) and cell cycle was monitored (Figure 2B) during cell expansion. We found that IPFSCs from both sgFN1a and sgFN1b groups grew faster than those from the copGFP group. This phenomenon was also supported by cell cycle data, in which both Cas9-sgFN1a and Cas9-sgFN1b transduced IPFSCs exhibited a higher percentage of cells in the S phase (%S). Our flow cytometry data suggested that FN1-KO decreased the expression of SSEA4 (Figure 2C) in both percentage and median and CD73 (Figure 2D) and CD90 (Figure 2E) in median but increased expression of CD105 (Figure 2F) in median and CD146 (Figure 2G) in both percentage and median in human IPFSCs.

To find out whether fibronectin affected stem cell stemness, a list of stemness genes was assessed using qPCR in FN1-KO IPFSCs and the control groups. Most stemness genes including *NANOG, SOX2, KLF4, BMI1*, and *MYC* were down-regulated in FN1-KO IPFSCs; however, some stemness genes, *NOV* and *POU5F1* (also known as *OCT4*), were down-regulated in the sgFN1a group but slightly up-regulated in the sgFN1b group (Figure 2H). Interestingly, *NES* was up-regulated in both sgFN1a and sgFN1b groups compared with the copGFP group (Figure 2H). We also found that all senescence-related genes were down-regulated in the FN1-KO IPFSCs, including *CDKN1A, CDKN2A*, and *TP53* (Figure 2I). Since fibronectin is linked with mesenchymal condensation, *CDH2*, a condensation marker, was also evaluated using qPCR. The data showed that *CDH2* expression in IPFSCs dramatically decreased in line with the extent of FN1-KO (Figure 2J).

### Effects of FN1-KO on IPFSCs in Chondrogenic and Adipogenic Differentiation

**Figure 3:**
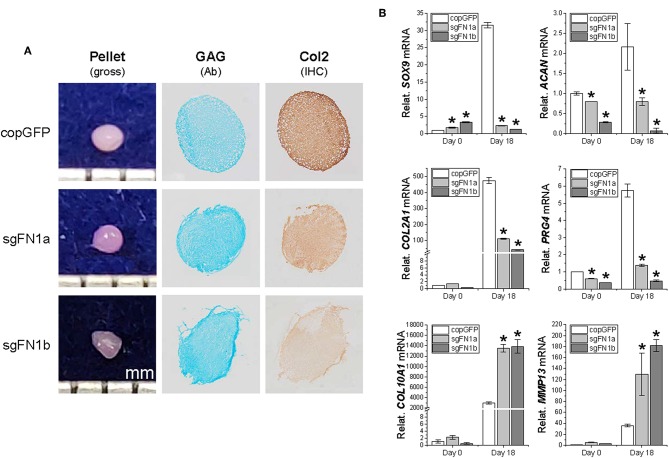
Chondrogenic potential of human IPFSCs after FN1-KO. Human IPFSCs were chondrogenically induced in a pellet culture system for 18 days. The effect of fibronectin on chondrogenic capacity of human IPFSCs was evaluated using gross observation of 18-day pellets, Alcian blue staining (Ab) for sulfated GAGs and immunohistochemical staining (IHC) for type II collagen (Col2) **(A)**. qPCR was used to evaluate expression of chondrogenic marker genes (*SOX9, ACAN, COL2A1*, and *PRG4*) and hypertrophic marker genes (*COL10A1* and *MMP13*) **(B)**. *GAPDH* was used as an endogenous control. Data are shown as bar charts. *indicates a significant difference compared to the corresponding copGFP group (*P* < 0.05).

**Figure 4:**
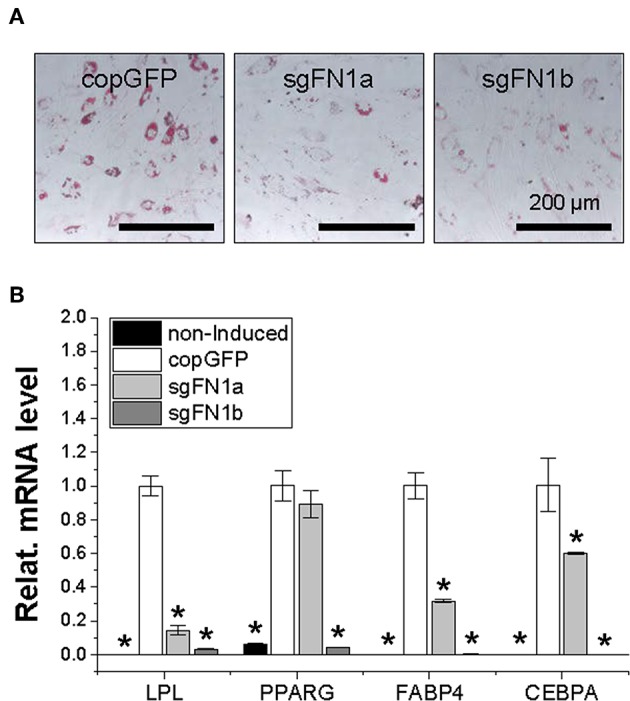
Adipogenic potential of human IPFSCs after FN1-KO. Human IPFSCs were adipogenically induced in differentiation medium for 21 days. The effect of FN1-KO on adipogenic capacity of human IPFSCs was evaluated using Oil Red O staining for liquid droplets **(A)** and qPCR for adipogenic marker gene (*LPL, PPARG, FABP4*, and *CEBPA*) expression **(B)**. *GAPDH* was used as an endogenous control. Data are shown as bar charts. *indicates a significant difference compared to the corresponding copGFP group (*P* < 0.05).

To ascertain whether FN1-KO affected IPFSCs' differentiation capacity, chondrogenesis (Figure 3) and adipogenesis (Figure 4) were evaluated using histology, immunostaining, and qPCR. A pellet culture system was employed for chondrogenic induction. After an 18-day chondrogenic incubation, FN1-KO IPFSCs yielded pellets with a smaller size and incomplete (rough) surface compared to the copGFP group, particularly for the sgFN1b group. This discrepancy was observed in Alcian blue staining for sulfated GAGs and IHC for type II collagen, two typical chondrogenic markers. The pellets from FN1-KO IPFSCs were weakly stained for both GAGs and type II collagen (Figure 3A); these results were validated at the mRNA levels by qPCR analysis demonstrating that FN1-KO significantly decreased the expression of chondrogenesis-related genes including *SOX9, ACAN, COL2A1*, and *PRG4* but increased hypertrophy-related genes in terms of *COL10A1* and *MMP13* (Figure 3B).

After a 21-day adipogenic induction, Oil Red O staining showed that, compared to the copGFP group, FN1-KO significantly decreased lipid droplets in induced IPFSCs, particularly for the sgFN1b group (Figure 4A). The staining data were further confirmed by qPCR data for typical adipogenic genes. The data showed that, compared to the copGFP group, the sgFN1a group had significantly lower expression of *LPL, FABP4*, and *CEBPA* but not *PPARG*, whereas the sgFN1b group had remarkably lower expression of all of the four adipogenic genes (Figure 4B).

### Impact of dECMs Deposited by FN1-KO Cells on IPFSCs in Proliferation and Stemness

**Figure 5:**
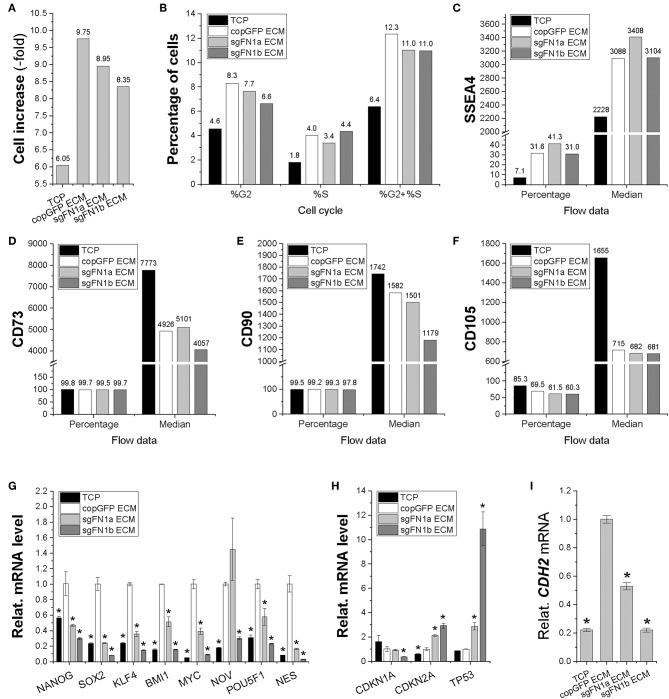
Proliferation capacity of human IPFSCs after expansion on the dECMs deposited by FN1-KO cells. Passage 15 human IPFSCs were compared after expansion on dECMs deposited by Cas9-sgFN1a/b transduced cells (sgFN1a ECM and sgFN1b ECM, respectively) with those deposited by copGFP (copGFP ECM) and those grown on TCP (TCP) as controls in cell increase **(A)**, percentage of cells in the S and G_2_ phases **(B)**, and surface markers [SSEA4 **(C)**, CD73 **(D)**, CD90 **(E)**, and CD105 **(F)**] by flow cytometry; stemness genes (*NANOG, SOX2, KLF4, BMI1, MYC, NOV, POU5F1*, and *NES*) **(G)**, senescence genes (*CDKN1A, CDKN2A*, and *TP53*) **(H)**, and the mesenchymal condensation gene (*CDH2*) **(I)** by qPCR. *GAPDH* was used as an endogenous control. Data are shown as bar charts. *indicates a significant difference compared to the corresponding copGFP group (*P* < 0.05).

In order to determine the influence of FN(–) matrix microenvironment on IPFSCs' proliferation, we compared passage 15 IPFSCs grown on sgFN1a ECM, sgFN1b ECM, copGFP ECM, and TCP. We found that all dECM groups exhibited higher cell increase (Figure 5A) and percentage of cells in the S and G_2_ phases (Figure 5B). Compared to the copGFP ECM group, a decline in cell growth and percentage of cells in the S and G_2_ phases was observed in the dECM groups deposited by FN1-KO IPFSCs. Our flow cytometry data also showed that, compared to the TCP group, all dECM-expanded cells exhibited increased expression of SSEA4 (Figure 5C) in both percentage and median but decreased expression of CD73 (Figure 5D) and CD90 (Figure 5E) in median and CD105 (Figure 5F) in both percentage and median, which were further strengthened in those grown on dECMs deposited by FN1-KO cells (Figures 5C–F).

To determine the effect of FN(–) matrix microenvironment on high passage IPFSCs' stemness, our qPCR data showed that, despite a dramatic up-regulation of all tested stemness genes including *NANOG, SOX2, KLF4, BMI1, MYC, NOV, POU5F1*, and *NES* in human IPFSCs grown on copGFP ECM compared to TCP, up-regulation of most stemness genes was diminished in IPFSCs after expansion on dECMs deposited by FN1-KO IPFSCs (Figure 5G). Interestingly, the FN(–) matrix microenvironment yielded expanded IPFSCs with higher expression of *CDKN2A* and *TP53*, but lower expression of *CDKN1A* (Figure 5H). Not surprisingly, compared to those grown on TCP, expansion on copGFP ECM yielded IPFSCs with up-regulation of *CDH2* expression; however, dECMs deposited by FN1-KO cells produced expanded cells with significantly lowered expression of *CDH2* (Figure 5I).

### Effects of dECMs Deposited by FN1-KO Cells on IPFSCs in Chondrogenic and Adipogenic Differentiation

**Figure 6:**
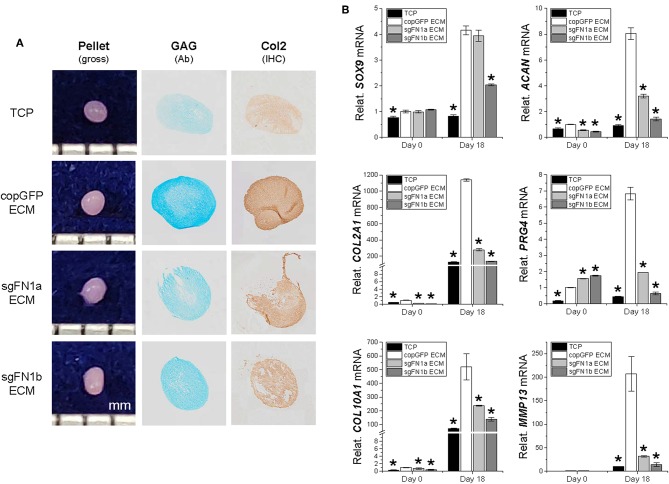
Chondrogenic potential of human IPFSCs after expansion on dECMs deposited by FN1-KO cells. Passage 15 human IPFSCs in a pellet culture system were compared for chondrogenic capacity after expansion on dECMs deposited by Cas9-sgFN1a/b transduced cells (sgFN1a ECM and sgFN1b ECM, respectively) with those deposited by copGFP (copGFP ECM) and those grown on TCP (TCP) as controls including gross observation of 18-day pellets, Alcian blue staining (Ab) for sulfated GAGs, and IHC for type II collagen (Col2) **(A)**. qPCR was used to evaluate expression of chondrogenic marker genes (*SOX9, ACAN, COL2A1*, and *PRG4*) and hypertrophic marker genes (*COL10A1* and *MMP13*) **(B)**. *GAPDH* was used as an endogenous control. Data are shown as bar charts. *indicates a significant difference compared to the corresponding copGFP group (*P* < 0.05).

We next wondered whether the FN(–) matrix microenvironment played a negative role in determining IPFSCs' differentiation preference. After chondrogenic induction, high passage IPFSCs grown on copGFP ECM yielded 18-day pellets with a larger size and more intensive staining of Alcian blue for sulfated GAGs and of IHC of type II collagen compared to those plated on TCP; however, this advantage of dECM expansion was diminished when high passage IPFSCs were expanded on dECMs deposited by FN1-KO cells (Figure 6A). These histological findings were supported by qPCR results showing that expansion on copGFP ECM yielded 18-day pellets with significantly higher expression of chondrogenic markers *SOX9, ACAN, COL2A1*, and *PRG4* as well as hypertrophic markers *COL10A1* and *MMP13*. However, expansion on dECMs deposited by FN1-KO cells yielded 18-day pellets with declining expression of these marker genes, particularly for the dECM deposited by Cas9-sgFN1b transduced cells (Figure 6B).

**Figure 7:**
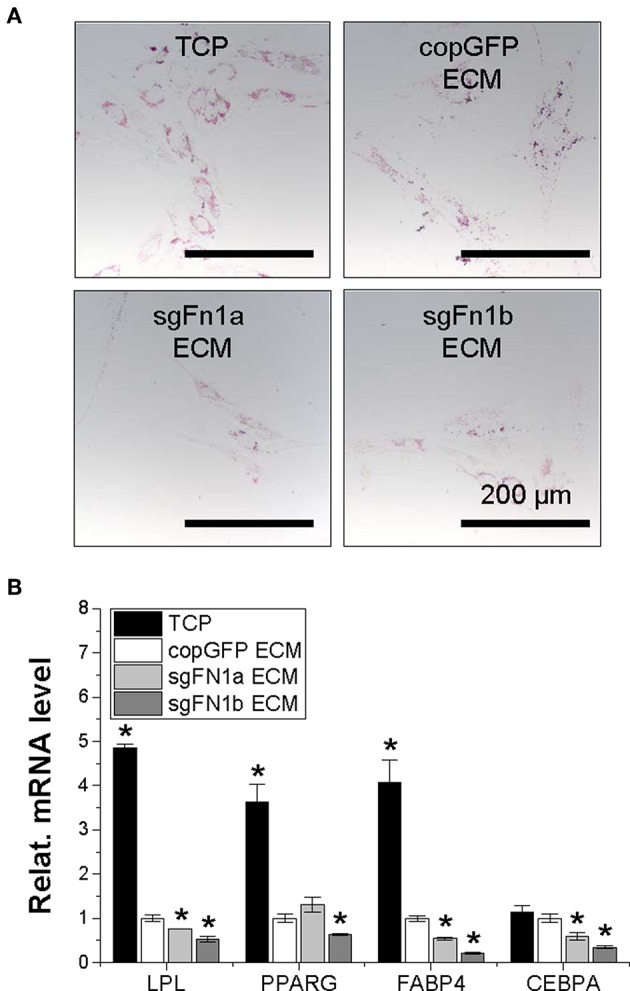
Adipogenic potential of human IPFSCs after expansion on dECMs deposited by FN1-KO cells. Human IPFSCs were compared for adipogenic capacity after expansion on dECMs deposited by Cas9-sgFN1a/b transduced cells (sgFN1a ECM and sgFN1b ECM, respectively) with those deposited by copGFP (copGFP ECM) and those grown on TCP (TCP) as controls including Oil Red O staining for lipid droplets **(A)** and qPCR for adipogenic marker gene (*LPL, PPARG, FABP4*, and *CEBPA*) expression **(B)**. *GAPDH* was used as an endogenous control. Data are shown as bar charts. *indicates a significant difference compared to the corresponding copGFP group (*P* < 0.05).

After adipogenic induction, we found that, compared to the TCP group, expansion on copGFP ECM yielded IPFSCs with less intensive staining of Oil Red O for lipid droplets, which further decreased if IPFSCs were pre-grown on dECMs deposited by FN1-KO cells (Figure 7A). This finding was consistent with qPCR data, in which expansion on TCP yielded IPFSCs with the highest expression of adipogenic marker genes *LPL, PPARG, FABP4*, and *CEBPA* after induction followed by expansion on copGFP ECM with the least expression in dECMs deposited by FN1-KO cells, particularly for the dECM deposited by Cas9-sgFN1b transduced cells (Figure 7B).

## Discussion

In this study, we used CRISPR/Cas9 technology to investigate the role of fibronectin in IPFSCs' proliferation and chondrogenic/adipogenic differentiation *via* direct FN1-KO in IPFSCs and indirect growth on dECMs deposited by FN1-KO IPFSCs. We found that FN1-KO increased IPFSCs' proliferation but decreased the proliferation of high passage IPFSCs grown on FN(–) dECM. Furthermore, FN1-KO had a negative effect on chondrogenic and adipogenic differentiation of IPFSCs, which was also reflected in high passage IPFSCs grown on FN(–) dECM.

The effect of fibronectin on stem cell proliferation remains controversial. Song et al. reported no effect of fibronectin on human BMSCs (Song et al., 2008); however, others reported that fibronectin promotes stem cell proliferation (Kalkreuth et al., 2014; Tao et al., 2018). In our study, we found that FN1-KO increased IPFSCs' proliferation, which was accompanied by up-regulation of CD105 and CD146 and down-regulation of senescence-associated genes. CD146, a putative surface marker of MSCs, is negatively linked with cellular senescence. For instance, CD146 expression was dramatically decreased in human umbilical cord blood-derived MSCs (UCB-MSCs) after long-term *in vitro* expansion; human UCB-MSCs with high CD146 expression exhibited a high rate of growth and telomerase activity as well as a notably lower expression of p16, p21, and p53 (Jin et al., 2016). Intriguingly, FN1-KO down-regulated most stemness genes except *NOV, POU5F1*, and *NES* in human IPFSCs. Interestingly, despite an increase of cell growth and percentage of cells in the S and G_2_ phases and SSEA4 expression in all dECM groups, expansion on dECMs deposited by FN1-KO cells yielded IPFSCs with decreased cell growth and cell cycling. Similar to previous reports (Zhang et al., 2015a,b; Pizzute et al., 2016), dECM expansion decreased expression of CD73, CD90, and CD105 in IPFSCs, which was strengthened in the sgFN1b ECM group. We also found that, compared to the TCP group, expansion on dECM deposited by copGFP cells exhibited the highest levels of stemness gene expression, which were diminished if grown on dECMs deposited by FN1-KO cells including *POU5F1* and *NES*. Interestingly, *CDKN2A* and *TP53* were up-regulated in IPFSCs after expansion on dECMs deposited by FN1-KO cells. Since few reports are available on interpretation of the interconnection among stem cells and surface markers and stemness gene expression, more research is needed to clarify the correlation.

Fibronectin promoted chondrogenic differentiation of mouse chondrogenic progenitor cells when fibronectin was included in the culture medium (Tao et al., 2018) and of human embryonic stem cells when cells were cultured on fibronectin type III domain-coated substrates (Cheng et al., 2018). Fibronectin matrix assembly was reported to be critical for cell condensation during chondrogenesis (Singh and Schwarzbauer, 2014). Given that fibronectin treatment could significantly decrease *COL10A1* and *MMP13* expression (Tao et al., 2018), it is reasonable to detect a dramatic increase of these two hypertrophic marker gene levels in FN1-KO IPFSCs. Considering that CDH2, a mesenchymal condensation marker, could enhance stem cell aggregation and subsequent chondrogenic differentiation (Goldring et al., 2006), decreased expression of *CDH2* in FN1-KO IPFSCs in our study might be responsible for the down-regulation of chondrogenic markers and incomplete surface of chondrogenic pellets.

In accord with previous reports (Pei, 2017), dECM-expanded high passage IPFSCs exhibited an enhanced chondrogenic differentiation, perhaps due to the sequestration of TGFβ in ECM (Horiguchi et al., 2012), which promoted dECM-expanded IPFSCs' stemness and amplified TGFβ-mediated chondrogenesis (Pei et al., 2011b). Similar to the appearance of pellets from FN1-KO IPFSCs, we found a rough exterior in the pellets of IPFSCs grown on dECMs deposited by FN1-KO cells, suggesting that human IPFSCs' differentiation preference is markedly influenced by FN(–) matrix microenvironment. It was found that deletion of fibronectin from young generating muscles reproduced the aging phenotype (Lukjanenko et al., 2016). Since a young environment contributed to an improved revitalization effect on aged progenitor cells (Conboy et al., 2005), it is reasonable to speculate a compromised rejuvenation effect of dECM resulted from FN1-KO related cell aging. Interestingly, inconsistent with up-regulated expression of hypertrophic markers in FN1-KO cells, IPFSCs plated on dECMs deposited by FN1-KO cells exhibited a down-regulated expression of hypertrophic marker genes. The underlying mechanisms remain unknown and deserve further investigation.

Despite adipogenesis being marked by a transformation from the fibronectin-rich stromal matrix (types I and III collagen, β1-integrin, and fibronectin) of the preadipocytes to the basement membrane (type IV collagen and entactin) of mature adipocytes (Gregoire et al., 1998; Selvarajan et al., 2001), we found that human IPFSCs after FN1-KO exhibited a dramatic decrease of adipogenic differentiation, as evidenced by Oil Red O staining for lipid droplets and qPCR for adipogenic marker genes, particularly for Cas9-sgFN1b transduced cells. This finding might be explained by the lack of fibronectin in human IPFSCs causing the failure of fibronectin fibrillogenesis, which is one of the crucial determinants for adipogenesis (Kamiya et al., 2002).

Many reports evaluated the effect of fibronectin on adipogenesis by using a fibronectin-coated surface or supplementing with fibronectin in the culture medium. For example, growth on fibronectin matrices inhibited adipogenesis of 3T3-F442A cells, which could be reversed by exposure to cytochalasin D that disrupted the actin cytoskeleton (Spiegelman and Ginty, 1983). Fukai et al. found that the addition of rat plasma fibronectin inhibited adipogenic differentiation of ST-13 preadipocytes; however, the thermolysin digest of fibronectin promoted adipocyte differentiation (Fukai et al., 1993). Considering potential sequestration and concentration of latent TGFβ by interaction with specific ECM components for future activation (Horiguchi et al., 2012), it seems reasonable that, compared to TCP culture, dECM expansion decreased IPFSCs' adipogenic differentiation because TGFβ is a potent inhibitor of adipogenic differentiation through promoting ECM synthesis including fibronectin (Gagnon et al., 1998). In this study, we also found that this unfavorable effect of dECM on adipogenic differentiation was further strengthened if dECMs were deposited by FN-KO cells. This effect might be explained by the influence of FN(–) matrix microenvironment on expanded human IPFSCs *via* mirror characters.

In summary, FN1-KO increased human IPFSCs' proliferation capacity; however, this capacity was reversed after expansion on dECMs deposited by FN1-KO cells. The importance of fibronectin in chondrogenic and adipogenic differentiation was demonstrated in both FN1-KO IPFSCs and the FN(–) matrix microenvironment, which might lay the foundation for fibronectin-mediated tissue engineering and regeneration.

## Data Availability Statement

The raw data supporting the conclusions of this manuscript will be made available by the authors, without undue reservation, to any qualified researcher.

## Author Contributions

YW: conception and design, acquisition of data, analysis and interpretation of the data, drafting the article, and final approval of the article. YF: acquisition of data, analysis and interpretation of the data, revising the article, and final approval of the article. ZY and X-BZ: conception and design, revising the article, and final approval of the article. MP: conception and design, analysis and interpretation of the data, drafting and revising the article, final approval of the article, and obtaining the funding.

### Conflict of Interest

The authors declare that the research was conducted in the absence of any commercial or financial relationships that could be construed as a potential conflict of interest.
